# Psychometric properties of the Group Climate Instrument (GCI) in individuals with mild intellectual disability or borderline intellectual functioning

**DOI:** 10.1111/jir.12567

**Published:** 2018-11-19

**Authors:** E. G. Neimeijer, J. J. Roest, G. H. P. van der Helm, R. Didden

**Affiliations:** ^1^ Radboud University, Behavioural Science Institute, Nijmegen and Trajectum Zwolle The Netherlands; ^2^ University of Applied Science, Youth Expert Centre Leiden The Netherlands

**Keywords:** borderline intellectual functioning, Group Climate Instrument, living group climate, mild intellectual disability

## Abstract

**Background:**

This study examined the psychometric properties of the Group Climate Instrument (GCI) in a sample of *N =* 189 adults (79% men) with mild intellectual disability or borderline intellectual functioning who were residents of a treatment facility in the Netherlands.

**Method:**

Construct validity of the GCI was examined by means of confirmatory factor analysis. Also, reliability and convergent validity of the GCI were examined. We also examined the variability in perception of the living group climate between and within living groups by computing intraclass correlation coefficients.

**Results:**

The model contained four first‐order factors (support, growth, group atmosphere and repression) and a second‐order factor overall climate, providing preliminary support for construct validity of the GCI. Reliability coefficients were good for all factors. Preliminary evidence for convergent validity was found in significant moderate associations between subscales and single item ratings for the factors of group climate. The intraclass correlation coefficients indicated that a considerate proportion of variance can be attributed to between‐group differences.

**Conclusions:**

The GCI might be used to assess perception of the living group climate for individuals with mild intellectual disability or borderline intellectual functioning in psychiatric and forensic care settings, although further development of the GCI and replication of our findings seem necessary.

## Background

There has been an extensive history of research into living group climate in (secure) residential settings for more than 50 years (Tonkin 2015). The notion that psychiatric and correctional units have a discernible social climate and the importance of such a climate have been recognised by the World Health Organization (1953) and Moos (1975). The World Health Organization stated that climate is ‘the most important factor in efficacy of treatment’ administered to psychiatric patients in prison and forensic psychiatric hospital settings, including individuals with intellectual disability (Tonkin 2015).

A therapeutic living group climate is essential for effective treatment in residential care (Schubert *et al*. 2012). Research shows that the quality of the living group climate has a positive influence on the development of clients (Van der Helm 2011). Transactional processes between sociotherapists 1Throughout this paper the term ‘sociotherapist’ is used to describe the role of professional caregiver. and clients and processes between clients make up most of the living group climate within the group (Van der Helm *et al*. 2011b). To create a therapeutic living group climate, sociotherapists should be responsive to fulfil basic psychological needs of the clients, such as the need for autonomy, competence and relatedness (Ryan and Deci 2017).

A distinction can be made between an open and a closed living group climate. An open living group climate is characterised by safety, mutual respect between clients and sociotherapists, structure in the day programme and prospects for growth and support to clients. Support provided by sociotherapists, which builds on meaningful relationships and responsivity to the needs of each individual client, sets the groundwork for successful rehabilitation (Andrews & Bonta 2010). An open living group climate has been shown to be associated with active coping, improved social information processing, empathy, prosocial behaviour, motivation for treatment, a longer period of treatment (no dropout) and higher levels of internal locus of control in participants (Lipsey 2009; Van der Helm *et al*. 2013; Van der Helm *et al*. 2014; Stams & Van der Helm 2016). An open living group climate is also associated with lower levels of aggressive and destructive behaviour of clients in secure care (Ros *et al*. 2013; De Decker *et al*. 2017).

A closed (i.e. repressive) living group climate, by contrast, is characterised by unfulfilled basic psychological needs as a result of rivalry, aggression, and insecurity among sociotherapists and clients and among clients on the living group. In such, climate sociotherapists are inclined towards restricting clients' autonomy, and excessive control instead of support, connectedness and flexibility towards clients. Furthermore, a closed living group climate is characterised by a lack of responsivity by sociotherapists, insufficient prospects for growth, an oppressive atmosphere in the living group and aggression among clients and among clients and sociotherapists (Harvey 2007). Also, a closed living group climate has negative consequences for the safety of both sociotherapists and clients (Van der Helm *et al*. 2011a).

Van der Helm *et al*. (2011a) provided an overview of climate research and found the same dimensions in a range of instruments measuring living group climate, namely, ‘support’, ‘growth’, ‘atmosphere’ and ‘repression’. These dimensions overlap with the basic psychological needs of clients (i.e. connectedness, autonomy and competence) from the perspective of the self‐determination theory (Ryan and Deci 2017). The dimension of ‘support’ represents the degree of sociotherapists' responsivity and support to clients. The clients' perception of support is based on connectedness, that is, the positive relationship between client and sociotherapists, whereas responsivity concerns the sociotherapists' response to the needs and characteristics of clients (i.e. feeling accepted, supported and included) (Van der Helm *et al*. 2011a; Ryan & Deci 2017). Research has shown that responsivity can be accomplished through offering support, stimulation of development, a trustworthy and respectful manner of treatment and consistency in procedures and sociotherapists' availability (Ackerman & Hilsenroth 2003; Marshall *et al*. 2003). ‘Growth’ concerns perceptions of learning and development and hope for the future and perceptions of the ability to feel competent and giving meaning to residing in the facility. Experiencing autonomy (i.e. exercising responsibility, choice and decision‐making) is essential for clients to be able to develop socially and emotionally (Van der Helm *et al*. 2011a; Ryan & Deci 2017). ‘Repression’ assesses perceptions of a lack of autonomy that threatens the basic psychological need of connectedness, competence and autonomy: strictness and control, unfair rules, lack of flexibility on the living group and boredom among clients (Harvey 2007; Van der Helm *et al*. 2009; Van der Helm *et al*. 2011a; De Valk *et al*. 2016). ‘Atmosphere’, finally, indicates the degree to which structure, security and trust among clients is fostered by both the physical and the social environment within the living group (Van der Helm *et al*. 2009; Van der Helm *et al*. 2011a).

As shown above, research highlights the importance of understanding living group climate in the light of effective treatment for individuals with mild intellectual disability or borderline intellectual functioning (MID‐BIF, Bressington *et al*. 2011; Schubert *et al*. 2012; Tonkin 2015). Although the relationship between living group climate and treatment outcomes is well researched and well documented in forensic and psychiatric services since Moos' early research in the 1960s, research into these outcomes for individuals with MID‐BIF is largely lacking (Moos 1989; Willets *et al*. 2014; Tonkin 2015; Bell *et al*. 2017). Tonkin (2015) shows that living group climate can be measured in a reliable and valid manner, and several instruments are available for measuring living group climate in clients without MID‐BIF. It is important that measurements are based on solid psychometric properties; otherwise, monitoring living group climate will hinder rather than help improving the quality of client care.

Measuring living group climate repeatedly and giving feedback to professionals working with these clients has been shown not only to improve living group climate but also to increase treatment motivation and empathy and diminish criminal cognitions which can facilitate return to society (Tonkin 2015). The Group Climate Instrument (GCI) was developed to measure the living group climate and has been proven to be a valid and reliable measure in other settings such as residential youth care, prisons and psychiatric (forensic) institutions and for different age groups (Van der Helm *et al*. 2011a). The majority of studies however have been undertaken with adolescents; therefore, the validity of the GCI with adults (with MID‐BIF) is less well established (Bell *et al*. 2017). To the best of our knowledge, no studies have been published on the psychometric characteristics of the GCI adapted to individuals with MID‐BIF in secure treatment facilities.

The aim of this study is to examine the psychometric properties (i.e. construct validity, convergent validity and internal consistency) of the GCI (Van der Helm *et al*. 2011a) in a sample of individuals with MID‐BIF (*N* = 189) who were residents of a secure treatment facility. As transactional processes between clients and sociotherapists and between clients make up a large part of the climate, we propose the perception of the living group climate to quality to be most salient in a living group level as opposed to individual or facility level. It is important that changes to working practices are made on the basis of the perspective of clients for which the GCI provides an important tool.

## Methods

### Participants

The sample of participants consisted of 189 participants, all residents of Trajectum, a (forensic) secure treatment facility for individuals with MID‐BIF located in the northern and eastern part of the Netherlands. All 441 residents were invited to participate in the study. In total, 208 residents participated (47% response rate). Data of 19 participants (9% of 208) were excluded from the analyses because of missing data of intellectual functioning (IQ), resulting in a sample of 189 participants. Participants (79% men) were aged between 18 and 69 years (M = 38.3, SD = 12.9). Of the participants, 44% had a mild ID (IQ 50–69) and 56% had borderline intellectual functioning (IQ 70–85) (M = 69.8, SD = 8.7). Besides MID‐BIF, participants had severe problem behaviour in combination with mental health problems and/or serious problems in all areas of life, often with a history of substance use. Comorbidity is high: most of the participants were diagnosed with more than one disorder, not including MID‐BIF. For most of the participants, the type of problem behaviour was the reason for admittance. Most of the participants were admitted because of externalising behaviour problems (i.e. aggression or a sexual offence) and/or internalising problems (like self‐injurious behaviour and suicide attempt) (Delforterie *et al*. 2018).

Participants were placed in the facility under criminal law or civil law. In the Netherlands, individuals who have committed a serious crime and are not legally accountable due to a mental disorder are sentenced by court to detention under hospital orders. This measure is not a punishment but an entrustment act for individuals with mental disorders which aims to protect society against the risk of recidivism trough incarceration and treatment. Treatment goals of participants placed under civil law include stabilisation and referral to regular mental health care. These participants are in need of intensive care in a secure setting due to severe behaviour and mental health problems, similar to participants placed under criminal law in terms of required intensity of treatment and level of security. The treatment duration in both cases is rarely shorter than 2 years and can last 10 years or more, depending on the participants' legal status and risk of (re)offending.

The facility consists of 58 living groups, and the modal ward size is eight beds. The mean treatment duration at Trajectum is 2 years and 4 months, although this is largely dependent on the participants' legal status and risk level. During treatment, participants move to wards with different levels of restrictions. Participants of this sample resided on wards which had different security levels; 59% of the participants resided on a low secure ward, 20% of the participants resided on a medium secure ward and 21% of the participants resided on a high secure ward.

### Procedure

Data were collected in the context of routine monitoring of the ward's climate within the facility. The study received approval from the local institutional review board. Each year, participants who resided in the facility were individually interviewed and completed the GCI. For the purpose of exploring psychometric properties of the instrument, only data from the first wave were used which were collected in March and April 2016. Participation was on a voluntary basis. The researcher gave oral and written information to participants concerning data collection, study aims and objectives. All participants and their legal guardians were informed that the research was strictly confidential and anonymous; data were only reported on a ward level and – upon approval – signed an informed consent form. In addition, the multidisciplinary treatment team determined whether a participant was able to give informed consent to participate. The active consent method was used; explicit consent was given by all of the participants. Questionnaires were given a code to guarantee anonymity of the participants. The names of participating participants were replaced by a code to ensure privacy.

Students of Windesheim University of Applied Sciences and (assistant) researchers of Trajectum were trained to conduct the questionnaire and signed a written statement of confidentiality. Most participants were assisted to complete the questionnaire by a student or (assistant) researcher who read the questions and answering categories out loud and explained the questions to the participant if necessary. Alternative scripted phrases to enable questions to be explained in a different way were part of the training they received. If used, this would provide an additional way of checking participants' understanding whilst preventing students and researchers from projecting their interpretation of the questions on to participants. The completed questionnaires were returned to the researcher (first author), after which the scores were entered into SPSS version 24 (IBM, SPSS Statistics) for analyses. Data on participant and context characteristics (gender, age, IQ, security level and legal status) were extracted from the records of the participants and added to the SPSS database.

### Group Climate Instrument

The quality of the living group climate was measured with the revised GCI which was adapted for individuals with MID‐BIF by using simpler wording compared to the original version (PGCI; Van der Helm *et al*. 2011a). The original version was reviewed for clarity, comprehensiveness, understanding, sensitivity and practical relevance during a brainstorm session with 10 young adults with MID‐BIF and a researcher. Based on this review, the questionnaire was shortened (from 36 items to 29 items), and items were reformulated/simplified. (e.g. ‘Sociotherapists listen to my opinion’ instead of ‘Sociotherapists pay attention to me and respect my feelings’ or ‘Sociotherapists help me when I ask them to’ instead of ‘When I have a problem, there is somebody I can turn to’). This resulted in a revised 29‐item questionnaire.

The GCI has four subscales: (1) support, (2) growth, (3) atmosphere and (4) repression. A total scale score is a combined score in which the subscale scores are added (after recoding of the items of the repression scale). The GCI was used to assess whether the living group climate is more open or more closed. The four factors are evident in both a closed and an open living group climate score. The balance between these two (i.e. open vs. closed) is decisive in terms of the quality of the climate. The outcomes produced by the GCI provide an in‐depth insight into living group climate from the clients' perspective and are being used to guide clinical practice and improve quality of client care.

The GCI consists of 29 items that can be scored on a Likert‐scale ranging from 1 (‘not applicable’) to 5 (‘entirely applicable’). The *Support* subscale contains 11 items and measures the responsivity of the sociotherapists towards the needs of participants, including giving attention to participants, taking complaints seriously and providing respect and trust. An example item of the growth subscale is ‘The sociotherapists treat me with respect’. The *Growth* subscale consists of six items and measures the degree to which participants feel they learn, gain hope for the future and comprehend the benefit of their stay at the ward. An example item of the growth subscale is ‘I learn the right things here’. The *Repression* subscale has seven items and measures the experience of strictness and control, unfair and coincidental rules and a lack of flexibility in the living group. An example item of the repression subscale is ‘You need to ask permission for everything here’. The *Atmosphere* subscale consists of five items and measures the degree to which participants trust one another, feel safe and secure towards one another (both clients and sociotherapists), are able to find rest and receive sufficient daylight. An example item of the atmosphere subscale is: ‘We trust one another here’.

In addition to filling out the GCI, participants were asked to evaluate the various factors of living group climate by giving a report mark (single item rating) between 1 (very poor) and 10 (excellent) to a statement, corresponding to the four subscales of the GCI. The statement ‘The support you receive from sociotherapists’ corresponded with the subscale support; the statement ‘What you learn here’ corresponded with the subscale growth; the statement ‘The atmosphere at the ward’ corresponded with the subscale atmosphere; and the statement ‘The rules at the ward’ corresponded with the subscale repression.

### Statistical analyses

Construct validity of the GCI was examined by means of confirmatory factor analysis (CFA). We used the lavaan package (Rosseel 2012) in the R environment (version 3.4.1; R Core Team 2017). A multifactor model was specified in which each item loaded on only one factor. The fit of the model was examined using the Comparative Fit Index (CFI), the Tucker–Lewis Index (TLI), Root Mean‐Square Error of Approximation (RMSEA) and the Standardised Root Mean Square Residual (SRMR). For a good‐fitting model, cut‐off values of CFI > .90, TLI > .90, RMSEA <.05 and SRMR < .08 are required (Hu & Bentler 1999; Kline 2005). We used the robust multiple linear regression maximum likelihood estimation procedure to account for non‐normality. A non‐significant chi‐square indicates exact model fit, a ratio between the *χ* ^*2*^ statistic and the degrees of freedom (*df*) lower than 2.5 indicates a close fit to the data (Hu & Bentler 1999). A modification index, giving the expected drop in chi‐square if the parameter in question is freely estimated, was used to improve model fit. Thus, parameters that could improve model fit by freeing those parameters were identified. Further improvement of model fit was achieved by removing one item that did not load significantly on the factor (one item of the repression scale).

Next, convergent validity was examined by calculating Pearson *r* correlations between the subscales of the GCI and the report marks (between 1 and 10). A positive moderate to strong correlation between the subscales support, growth and atmosphere and the corresponding report marks is seen as indicative of convergent validity of the three subscales. A negative moderate to strong correlation between the repression subscale and the corresponding report mark for repression indicates convergent validity of the subscale repression. Pearson's correlations of *r =* .10–.30 are seen as small, *r =* .30–.50 are seen as a moderate and *r* > .50 are seen as a large (Cohen 1988). Reliability analyses were conducted in SPSS 24 (both Cronbach's alpha and Guttman's Lambda‐2). Alpha's above .70 and .79 were fair; between .80 and .89 were good (Cicchetti 1994). For interpreting reliability estimates, including Guttman's lambda‐2 (λ‐2), there are some general rules of thumb; λ‐2 above .70 are sufficient for group‐level studies (Guttman 1945; Osburn 2000). In order to determine what proportion of the variance in each of the four living group climate subscales could be attributed to the group level and the individual level, we computed the intraclass correlation coefficient (ICC) which is calculated by dividing the level‐2 variance by the total variance (Raudenbush & Bryk 2002). Items with an ICC close to zero indicate that variation is mainly within clients, instead of between living groups. On the other hand, items with an ICC that approximates 1 indicate that variation is mainly between living groups, instead of within clients. Because our goal was to measure living group climate, a group construct, it is important to examine the variance of scores at the between‐group level.

## Results

Results for the GCI indicated a good fit to the data. CFA was conducted on all 29 GCI items. Results showed factor loadings ranging from .234 to .828 (Table 1). The model showed an acceptable fit to the data: *χ* ^2^(334) = 457.152 (*P* < .001); CFI = .931; TLI = .922; RMSEA = .048 (90% CI = .036–.058); SRMR = .071. The ratio between the *χ* ^2^ statistic and the degrees of freedom was 1.37. One item of the repression subscale (i.e. ‘Clients must ask permission for everything’) did not load significantly on the repression factor and was deleted from the model to improve model fit. Further analyses were conducted with 28 items. Table 1 presents the final factor solution, showing the items and the corresponding factor loadings. The model that best fitted the data contained four first‐order factors [support (11 items), growth (6 items), group atmosphere (6 items) and repression (5 items)] and a second‐order factor ‘overall climate’.

**Table 1:** Standardised regression weights of the Group Climate Instrument (28 items)

Item no.	Subscale/item	Standardised estimates for first order factors	Standardised estimates for second order factor
	Support		.956
2	Sociotherapists help me when I ask them to.	.726	
5	I trust the sociotherapists.	.760	
6	I think the sociotherapists are honest.	.814	
7	I get attention from the sociotherapists.	.712	
8	The sociotherapists listen to my opinion.	.697	
17	Because of the sociotherapists, I try new things.	.578	
18	When I have a complaint, it will be dealt with.	.570	
22	There are always enough people around to help me.	.538	
24	The sociotherapists have little time for me.	−.390	
25	I think the sociotherapists deal with angry clients in a good way.	.587	
26	The sociotherapists often talk things through with the clients.	.651	
	Growth		.806
11	I work on my goals here.	.603	
12	I think it is good that I'm here.	.657	
13	Here, I learn how to behave outside the institution.	.674	
16	I get to decide things for myself here.	.309	
19	What I learn here helps me.	.826	
21	I learn the right things here.	.793	
	Repression		−.722
15	The sociotherapists always get their way.	.234	
20	I'm bored here.	.557	
23	I feel understood by the sociotherapists.	−.671	
27	There is nothing to do here.	.330	
28	It is dirty and it smells bad here.	.396	
29	This ward makes me feel down.	.824	
	Atmosphere		.820
1	There is a good atmosphere on the ward.	.571	
4	I feel good at the ward.	.818	
9	The turmoil on the ward drives me crazy.	−.305	
10	On the ward, clients trust each other.	.524	
14	It is safe on the ward.	.828	

To examine convergent validity of the GCI, Pearson's *r* was used to calculate correlations between the subscales of the GCI and the report marks (between 1 and 10). We found that repression had a negative correlation with the statement ‘the rules at the ward’ (*r* = −.339, *P* < .01). Positive correlations were found between support and ‘the support you receive from sociotherapists’ (*r =* .681; *P* < .01), growth and ‘what you learn here’ (*r =* .666; *P* < .01) and atmosphere and ‘the atmosphere at the ward’ (*r =* .663; *P* < .01) (Table 2). All correlations are moderate to strong and were in the expected direction which may be seen as supportive of convergent validity.

**Table 2:** Correlations between subscale scores and report marks

Report mark	Subscale support	Subscale growth	Subscale repression	Subscale atmosphere
‘The support you receive from sociotherapists’	.681**	.584**	−.361**	.513**
‘What you learn here’	.542**	.666**	−.248**	.450**
‘The rules at the ward’	.356**	.407**	−.339**	.477**
‘The atmosphere at the ward’	.498**	.401**	−.293**	.663**

** *P* < .01

The GCI was found to be internally consistent, with alpha's ranging between .642 and .882. The ICCs for the group climate subscales and the total climate scale ranged from .193 to .385. These results indicate that a considerate proportion of variance (roughly 19–39%) can be attributed to between‐group differences (i.e. the group level). Means, standard deviations, ICCs and results of reliability coefficients in terms of Cronbach's alpha (α) and Guttman's Lambda‐2 (λ‐2) are displayed in Table 3.

**Table 3:** Results of the reliability analyses of the Group Climate Instrument (28 items)

Subscale	λ‐2	α	M	SD	ICC
Support	.884	.882	3.668	0.815	.279
Growth	.795	.786	3.809	0.971	.300
Repression	.662	.642	2.883	0.727	.193
Atmosphere	.770	.762	3.283	0.808	.340
Overall climate	.922	.918	3.556	0.754	.385

ICC, intraclass correlation coefficient.

## Discussion

The aim of this study was to explore the psychometric properties of the GCI for individuals with MID‐BIF who resided in a (forensic) secure treatment facility. We used conventional single level CFA to examine the factor structure of the GCI. The present study provides preliminary evidence for the construct validity and reliability of the GCI for individuals with MID‐BIF. Results showed an adequate fit for a first‐order and second‐order model, which indicates construct validity of the GCI. Reliability coefficients for all scales were satisfactory. The support subscale loaded highest on the overall group climate scale, which indicates that support is the most important indicator of group climate for individuals with MID‐BIF. One item of the repression subscale (i.e. ‘Clients must ask permission for everything’) did not load significantly on the repression factor as a result of which it was deleted from the model to improve model fit. This finding may be related to the fact that the repression subscale had relatively lower loadings on the overall climate scale but also to the heterogeneity among the items in order to adequately capture the multifaceted nature of the construct (Van der Helm *et al*. 2011a; Heynen *et al*. 2014; De Valk *et al*. 2016). The finding that this item is unsuitable to measure repression cannot be explained by current research. The ICCs found in the present study indicated that a substantial portion of variance can be attributed to the between‐group level. In other words, the living group in which each client resided accounted for a considerate proportion of the variability in perception of living group climate. Our results indicate that the perception of the clients who reside in the same group is more similar to each other compared to clients from different groups. Multilevel analyses are recommended to explain between‐group variance.

There are several limitations of this study that need to be acknowledged. First, although the main aim of the study was to assess construct validity and reliability of the GCI for individuals with MID‐BIF, client and other characteristics may be differentially related to (sub)scale scores of the GCI. Future studies should examine possible differences in perceived living group climate between different subgroups, addressing within‐group (IQ, diagnosis, age, gender, legal status, criminal history, etc.) and between‐group (security level, ward size, intensity of support, etc.) variables. It cannot be ruled out that the participants did not understand some of the questions. However, because there were no dropouts and no missing data, we believe this did not influence our results. In order to keep the level of interviewing as high as possible, monthly meetings were organised to align with all interviewers how to present and explain information to participants unambiguously. Neither the possibility of socially desirable answers can be excluded. Consistency in answering patterns, the fact that the questionnaire contains both positively and negatively formulated items and interviewers were not in any way involved in treatment, suggests that the influence of social desirability was minimised.

A further limitation is that we used a single item measure to assess convergent validity of the GCI. This may yield biased results, because the statements corresponding to the subscales may not capture all relevant aspects of the different factors of living group climate. Future studies should assess convergent validity of the GCI with a validated GCI, such as the EssenCES (De Vries *et al*. 2018). Also, future studies should examine concurrent validity and predictive validity of the GCI in populations with MID‐BIF. Concurrent validity can be assessed by relating group climate to aggressive behaviour during treatment, such that a positive group climate could be associated with fewer aggressive incidents (Ros *et al*. 2013; De Decker *et al*. 2017). Predictive validity can be established by examining the relationship between quality of the living group climate and treatment outcomes (Bressington *et al*. 2011; Schubert *et al*. 2012; Tonkin 2015).

Another important methodological limitation is that we used conventional single level CFA to examine the factor structure of the GCI. The ICCs found in the present study indicate that a substantial portion of variance can be attributed to the between‐group level. Therefore, multilevel analysis is warranted (Hox 2002; Hahs‐Vaughn 2016). The assumption is that the perception of group climate varies across individuals, and groups vary in average level of group climate. Also, it can be argued that the perception of the living group climate is determined by characteristics of the living group more strongly than characteristics of participants. An important advantage of multilevel confirmatory factor analysis is that the factor structure of a measure can be examined at both the within‐group level and the between‐group level (Huang 2017). However, in the present study, the sample size was insufficient to conduct a multilevel confirmatory factor analysis. Future studies on the GCI (and other GCIs, see Tonkin 2015) should focus on the clustered nature of group climate measures (individuals are nested within living groups). It is important to examine the factor structure of the GCI at both the within‐group level and between‐group level, to test whether the factor structure of the GCI is the same at both levels. Future research on the factor structure and reliability of the GCI at the between‐group level is important to assess construct validity of the GCI.

## Conclusion

The present study is the first study that examined psychometric properties of the GCI adapted to measure perceived living group climate in individuals with MID‐BIF and severe behavioural problems. The GCI could be used to monitor the living group climate in secure forensic facilities for individuals with MID‐BIF on a regular basis. That contributes to our understanding of how the living group climate can be improved for the benefit of both sociotherapists and clients with MID‐BIF in secure settings. The current findings are found not only in secure residential care for children, adolescents and adults without MID‐BIF (Van der Helm *et al*. 2011b; Heynen, *et al*. 2014, Strijbosch *et al*. 2014) but also in residential care and treatment for adults with MID‐BIF. These outcomes also brings us a step closer to a standardised instrument that can be used to measure living group climate in different kinds of settings and in a broader range of target groups and to evaluate the effectiveness of interventions that aim to improve living group climate.

## Ethics approval

Ethics approval was granted from the Ethics Committee of the Faculty of Social Sciences (ECSS) of the Radboud University (ECSW2017‐3001‐471).

## Conflict of Interests

The authors declared no potential conflicts of interest with respect to the research, authorship and/or publication of this article.

## Source of funding

This research was funded in part by aNWOSia‐Raak grant: ‘Met kleine stapjes maar wel vooruit’.
